# Nahrungsergänzungsmittel im Kontext sozialer Medien: Ergebnisse einer Befragung zur Nutzung und Wahrnehmung in Deutschland

**DOI:** 10.1007/s00103-025-04133-2

**Published:** 2025-10-01

**Authors:** Henri Obstfeld, Mark Lohmann

**Affiliations:** Abteilung Risikokommunikation, Bundesinstitut für Risikobewertung, Max-Dohrn-Str. 8–10, 10589 Berlin, Deutschland

**Keywords:** Mikronährstoffe, Risikowahrnehmung, Motivation, Informationsverhalten, Risikokommunikation, Micronutrients, Risk perception, Motivation, Information behaviour, Risk communication

## Abstract

**Hintergrund:**

In den letzten Jahren haben Nahrungsergänzungsmittel (NEM) wie Vitamin- und Mineralstoffpräparate stark an Popularität gewonnen. Dies zeigt sich auch in sozialen Medien, wo insbesondere Influencerinnen und Influencer Informationen zu NEM verbreiten oder entsprechende Produkte bewerben. Verbraucherinnen und Verbraucher erhalten so möglicherweise einen einseitigen Eindruck von NEM. Da die Einnahme von NEM sowohl spezifische gesundheitliche Nutzen als auch Risiken bergen kann, ist eine zielgruppengerechte Aufklärung notwendig. Grundlage hierfür sind Kenntnisse über Wahrnehmung und Verwendung von NEM in der Bevölkerung.

**Methoden:**

In einer (hinsichtlich Alter, Geschlecht, Schulbildung und Bundesland) repräsentativen Online-Befragung (*n* = 1071) wurden im September 2024 Nutzungsverhalten, Einnahmemotivation und Wahrnehmung hinsichtlich NEM in Deutschland untersucht.

**Ergebnisse:**

Von den Befragten gaben 76,6 % an, im vergangenen Jahr NEM eingenommen zu haben, vor allem spezifische Mikronährstoffe wie Magnesium und Vitamin D. Hauptgründe für die Verwendung waren gesundheitsbezogene Motive. Zudem schätzten 14,3 % der Verbraucherinnen und Verbraucher NEM der rechtlichen Definition entsprechend als Lebensmittel ein. Je nachdem, ob Befragte Informationen zu NEM in sozialen Medien wahrgenommen hatten oder nicht, zeigten sich Unterschiede in Nutzungsverhalten, Einnahmemotivation und Wahrnehmung von NEM. Personen, die Informationen zu NEM aus sozialen Medien erhalten hatten, nahmen eine größere Zahl unterschiedlicher Stoffe ein, verwendeten NEM eher mindestens wöchentlich und bewerteten deren Nutzen höher.

**Diskussion:**

Die Ergebnisse zeigen sowohl Implikationen für die Gesundheits- und Risikokommunikation als auch den Bedarf für weitere Forschung auf.

## Abstract (en)

**Background:**

In recent years, food supplements such as vitamin or mineral preparations have gained great popularity. This is also evident on social media where influencers in particular disseminate information about or advertise food supplements. This way, consumers may get a one-sided impression of food supplements. As the intake of food supplements is associated with specific health risks and benefits, target group-specific education is necessary. The foundation for this is knowledge of perceptions and use of food supplements in the population.

**Methods:**

A representative (for age, gender, education and federal state) online survey (*n* = 1071) was conducted in September 2024 to investigate usage behaviour, intake motivation, and perceptions with regard to food supplements in Germany.

**Results:**

Of the respondents, 76.6% reported having taken food supplements during the past year, particularly specific micronutrients such as magnesium or vitamin D. The main reasons for their usage were health-related. In addition, 14.3% of consumers considered food supplements as food in accordance with the legal definition. There were differences in usage behaviour, intake motivation and perception of food supplements depending on whether or not respondents had perceived information about food supplements on social media. Persons who had received information about food supplements on social media took a greater number of different substances, were more likely to take food supplements at least once a week and rated their benefit higher.

**Discussion:**

The findings reveal both implications for health and risk communication and the need for further research.

## Hintergrund

Die Einnahme von Nahrungsergänzungsmitteln (NEM) ist weitverbreitet. So lassen aktuelle Befragungen darauf schließen, dass in Deutschland die Hälfte bis 3 Viertel der Menschen NEM kaufen bzw. einnehmen [1, 2].

Als NEM werden Produkte definiert, die bestimmte Mikronährstoffe oder weitere Inhaltsstoffe mit ernährungsspezifischer oder physiologischer Wirkung in konzentrierter Form, z. B. als Tablette oder Pulver, zur gezielten Ergänzung der Ernährung beinhalten [3]. Eine ausreichende Versorgung mit Mikronährstoffen wie Vitaminen und Mineralstoffen ist aufgrund ihrer Funktionen im Stoffwechsel essenziell [4]. In bestimmten Fällen eignen sich NEM, um eine ausreichende Nährstoffzufuhr sicherzustellen. Dabei ist besonders zu beachten, dass neben Risiken durch eine Unterversorgung auch Risiken durch eine Überversorgung bestehen [5]. So kann beispielsweise eine hoch dosierte Einnahme von Vitamin D oder Omega-3-Fettsäuren mit gesundheitlichen Beeinträchtigungen wie einer Verringerung der Knochendichte oder Vorhofflimmern verbunden sein [6, 7]. Eine Nahrungsergänzung ist angesichts der gesundheitlichen Nutzen und Risiken, die zudem zwischen Bevölkerungsgruppen variieren können, individuell abzuwägen. Da das Angebot an NEM vielfältig und expandierend ist, besteht die Notwendigkeit, die Bevölkerung auf gesundheitliche Auswirkungen einer übermäßigen Nährstoffzufuhr, insbesondere durch NEM, aufmerksam zu machen.

Obwohl sie in ihrer Darreichungsform und Vermarktung teilweise Arzneimitteln ähneln, gelten NEM rechtlich als Lebensmittel [3] und werden entsprechend reguliert. Das bedeutet beispielweise, dass anders als bei freiverkäuflichen Arzneimitteln die Verantwortung für die Sicherheit und Wirksamkeit von NEM bei den Herstellern liegt und keine Zulassung und Prüfung durch eine unabhängige Instanz erfolgen [8]. Als Lebensmittel können NEM unter anderem in Supermärkten, Drogerien und im Onlinehandel niedrigschwellig erworben werden. Die Einnahme von NEM erfolgt somit häufig ohne ärztliche Absprache [9]. Inwiefern der Bedarf an NEM medizinisch erforderlich ist, bleibt dabei in der Regel ungeklärt.

Bei der Verbreitung und Rezeption von Informationen zu NEM gewinnen soziale Medien, die auch als Plattform für Sport- und Ernährungsinhalte dienen, zunehmend an Bedeutung. Die Inhalte werden unter anderem von Influencerinnen und Influencern erstellt, die sich durch eine große Reichweite auszeichnen und über ihre Inhalte Einfluss auf ihr Publikum nehmen [10, 11]. Insbesondere zu Themenbereichen wie Fitness und Ernährung werden sie als Informationsquelle herangezogen [12, 13] und auch ihr Einfluss auf Kaufentscheidungen nimmt zu [14]. Entsprechend haben sich Influencerinnen und Influencer zu einem etablierten Marketingkanal für Unternehmen entwickelt – auch für Hersteller und Händler von NEM. Bei Influencerinnen und Influencern im Bereich Sport und Ernährung zählen NEM zu den am häufigsten beworbenen Produktkategorien [15].

Die Vermarktung von NEM beinhaltet oft nährwert- oder gesundheitsbezogene Angaben, deren Verwendung bei der Werbung für Lebensmittel wie NEM über die europäische Health-Claims-Verordnung (HCVO) geregelt ist [16]. Die Einhaltung dieser Regelung durch Werbetreibende in den sozialen Medien erweist sich jedoch oftmals als lückenhaft. So zeigt eine Untersuchung von Beiträgen in sozialen Medien, dass etwa 39 % der von Lebensmittelunternehmen getätigten gesundheitsbezogenen Aussagen nicht zulässig waren [17]. Speziell Influencerinnen und Influencer verstießen sogar in etwa 90 % der untersuchten gesundheitsbezogenen Aussagen gegen die HCVO. Zugleich gibt es Belege, dass gesundheitsbezogene Aussagen über Lebensmittel Einfluss auf die Produktwahrnehmung von Verbraucherinnen und Verbrauchern haben [18–21]. Es ist daher denkbar, dass sich die Exposition mit Werbebeiträgen in sozialen Medien auch auf die Risiko-Nutzen-Wahrnehmung von NEM auswirkt.

Vor diesem Hintergrund untersucht die aktuelle Befragungsstudie die Nutzung, Wahrnehmung und Einnahmemotivation hinsichtlich NEM in der Bevölkerung in Deutschland. Angesichts der verstärkten Bewerbung von NEM in sozialen Medien wird darüber hinaus eruiert, ob es diesbezüglich Unterschiede zwischen Verbraucherinnen und Verbrauchern gibt, je nachdem, ob sie entsprechende Informationen über soziale Medien erhalten oder nicht. Damit soll die Grundlage für eine adäquate Risikokommunikation zu NEM geschaffen werden.

## Methodik

### Studiendesign, Stichprobe und Datenerhebung

Für die Umsetzung dieser Studie wurde eine Online-Befragung (CAWI) durchgeführt. Die Grundgesamtheit bildete die deutschsprachige und internetnutzende Wohnbevölkerung ab 16 Jahren in Deutschland. Die Stichprobe wurde über 2 einladungsbasierte Online-Access-Panels in Bezug auf Alter, Geschlecht, Bildung und Bundesland geschichtet gezogen. Um Abweichungen von der Zielverteilung zu korrigieren, wurden die Daten für diese soziodemografischen Variablen entsprechend der Verteilung in der Grundgesamtheit gewichtet.

Insgesamt nahmen 1156 Personen vom 09.09.2024 bis 19.09.2024 an der Befragung teil. Nach Ausschluss von 85 Fällen mit auffälligem Antwortverhalten^1^ wurde in den Analysen eine Stichprobe von *n* = 1071 Befragten verwendet (Tab. 1). Die Fehlermarge als Maß für die statistische Unsicherheit beträgt 3 % (bei Konfidenzniveau: 95 %, Anteilswert: 50 %).

**Tab. 1:** Demografische Verteilung für die Gesamtstichprobe, NEM-Nutzende und SoMe-Informierte

		Gesamt			NEM-Nutzende	SoMe-Informierte
		Ungewichtet		Gewichtet	Gewichtet	Gewichtet
		*n*	In Prozent	In Prozent	In Prozent	In Prozent
Geschlecht	Männlich	517	48,3	49,6	46,7	46,4
	Weiblich	547	51,1	49,6	52,6	52,8
	Divers	7	0,7	0,7	0,6	0,9
Alter	16–39	354	33,1	36,6	39,0	49,9
	40–59	403	37,6	34,7	34,5	32,7
	60+	314	29,3	28,7	26,4	17,3
Schulbildung	Niedrig	269	25,1	29,7	27,4	20,7
	Mittel	372	34,7	30,7	31,4	32,6
	Hoch	419	39,1	38,6	40,5	46,2
	Andere	11	1,0	1,1	0,7	0,5

Gesamt: *n* = 1071, NEM-Nutzende: *n* = 824, SoMe-Informierte: *n* = 486 (Fallzahlen ungewichtet)

*NEM* Nahrungsergänzungsmittel, *SoMe* soziale Medien

Die Datenerhebung wurde von einem Markt- und Sozialforschungsinstitut durchgeführt. Die Teilnahme an der Befragung war freiwillig, erfolgte nach Einverständnis der Befragten und konnte jederzeit beendet werden. Die erhobenen Daten lassen keinen Rückschluss auf einzelne Personen zu.

### Fragebogen

Neben soziodemografischen Angaben umfasste der Fragebogen Fragen zu den Themenbereichen Nutzungsverhalten, Einnahmemotivation, Wahrnehmung und Informationsverhalten in Bezug auf NEM sowie allgemeines Ernährungs- und Gesundheitsverhalten. Die durchschnittliche Befragungsdauer betrug nach Fallausschluss 21 min. Zur Optimierung des Fragebogens hinsichtlich der Verständlichkeit und Funktionalität wurde vorab mit *n* = 103 Personen ein Pretest durchgeführt.

Um die Einnahmeprävalenz von NEM zu bestimmen, wurden die Befragten zu insgesamt 61 Vitaminen, Mineralstoffen, pflanzlichen und weiteren Inhaltsstoffen gefragt, ob sie diese in den letzten 12 Monaten über NEM eingenommen haben. Befragte, die nach eigener Angabe mindestens einen der abgefragten Stoffe in den vergangenen 12 Monaten eingenommen haben, wurden als NEM-Nutzende definiert. Darüber hinaus wurden für NEM allgemein die Einnahmehäufigkeit (6-stufige Skala von 1 = „täglich“ bis 6 = „gar nicht“) und die Wahrscheinlichkeit einer zukünftigen Einnahme von NEM, die bisher noch nicht eingenommen wurden (5-stufige Skala von 1 = „sehr unwahrscheinlich“ bis 5 = „sehr wahrscheinlich“), erhoben.

Zur Erfassung der Wahrnehmung von NEM wurden die Befragten gebeten, 5 Aussagen zu NEM (z. B. „Nahrungsergänzungsmittel sind freiverkäufliche Arzneimittel“) auf einer 5‑stufigen Skala von 1 = „trifft gar nicht zu“ bis 5 = „trifft voll und ganz zu“ zu bewerten.

Um die Einnahmemotivation zu erheben, gaben NEM-Nutzende für 26 Gründe auf einer 5‑stufigen Skala (1 = „trifft gar nicht zu“ bis 5 = „trifft voll und ganz zu“) an, inwiefern diese auf ihre eigene Einnahme zutreffen (z. B. „Ich nehme Nahrungsergänzungsmittel ein, weil ich meinen aktuellen Gesundheitszustand erhalten möchte“). Dabei wurden neben gesundheitsbezogenen unter anderem auch defizitbezogene und soziale Motive abgefragt. Darüber hinaus wurden die NEM-Nutzenden gebeten, aus 26 konkreten Anwendungsbereichen (z. B. Haut, Haare und Nägel oder Stärkung der Muskulatur) die für sie zutreffenden auszuwählen.

Zur Erfassung der Risiko- und Nutzen-Wahrnehmung wurden die Befragten gebeten, auf einer 5‑stufigen Skala von 1 = „sehr niedrig“ bis 5 = „sehr hoch“ jeweils das Risiko und den Nutzen einer selbstständigen Einnahme von freiverkäuflichen NEM ohne eine vorherige Beratung durch einen Arzt oder eine Ärztin einzuschätzen.

Die subjektive Informiertheit zu NEM wurde mit 5 Items erhoben, für die die Befragten angaben, wie gut sie sich über die jeweiligen Aspekte von NEM (z. B. gesundheitliche Risiken) informiert fühlen (5-stufige Skala von 1 = „sehr schlecht“ bis 5 = „sehr gut“).

Hinsichtlich des Informationsverhaltens über soziale Medien wurde gefragt, wie häufig über soziale Medien oder von Influencerinnen und Influencern werbliche oder nichtwerbliche Informationen zu NEM wahrgenommen werden (5-stufige Skala von 1 = „nie“ bis 5 = „sehr häufig“). Als Gruppe der Soziale-Medien-Informierten („SoMe-Informierte“) wurden solche Befragten definiert, die mindestens gelegentlich werbliche oder nichtwerbliche Informationen zu NEM von Influencerinnen oder Influencern oder über soziale Medien wahrnehmen. Die restlichen Befragten, die angaben, keine sozialen Medien zu nutzen oder seltener als gelegentlich von Influencerinnen oder Influencern oder über soziale Medien Informationen zu NEM zu erhalten, wurden als Gruppe der Nicht-soziale-Medien-Informierten („Nicht-SoMe-Informierte“) definiert.

Zusätzlich wurden SoMe-Informierte nach Plattformen, Art der wahrgenommenen Informationen (z. B. Informationen über Vorteile) und fachlichem Hintergrund der wahrgenommenen Influencerinnen und Influencern gefragt.

### Statistische Analyse

Die Datenaufbereitung erfolgte mit SPSS (IBM Corp., Armonk, NY, USA; Version 26; [22]), die statistische Analyse der gewichteten Daten wurde mit dem Survey-Paket in R (R Foundation for Statistical Computing, Wien, Österreich; Version 4.4.1; [23, 24]) durchgeführt. Für Vergleiche zwischen NEM-Nutzenden und Nichtnutzenden sowie SoMe-Informierten und Nicht-SoMe-Informierten wurden Χ^2^- und t‑Tests durchgeführt. Als Effektstärken wurden Cohens d und Cramers V berechnet, die mit dem Effectsize-Paket nach Cohen interpretiert wurden [25, 26].

## Ergebnisse

### Nutzungsverhalten

Insgesamt gaben 76,6 % der Befragten an, in den letzten 12 Monaten mindestens einen der abgefragten Inhaltsstoffe über NEM eingenommen haben. Durchschnittlich gaben diese Nutzenden an, 7,8 (SD = 7,8) verschiedene Inhaltsstoffe über NEM eingenommen zu haben. Insbesondere einzelne Vitamine und Mineralstoffe wurden von vielen Befragten eingenommen. Dabei sind Magnesium, Vitamin D und Vitamin B12 die am häufigsten eingenommen Stoffe (Tab. 2).

**Tab. 2:** Prävalenz für die Einnahme von Mikronährstoffen und weiteren Inhaltsstoffen über NEM sowie Gruppenvergleiche nach SoMe-Informiertheit; Darstellung von Mikronährstoffen und weiteren Inhaltsstoffen, die von über 10 % aller Befragten eingenommen werden

	Häufigkeiten in Prozent			Signifikanz	Effektstärke
Stoff	Gesamt	SoMe-Informierte	Nicht-SoMe-Informierte	*p*	V
Magnesium	53,6	53,7	53,6	0,960	0,00
Vitamin D	40,1	42,7	37,9	0,120	0,05
Vitamin B12	34,0	37,7	31,0	0,025	0,07
Vitamin C	31,6	33,6	30,0	0,225	0,04
Zink	25,6	28,8	23,0	0,033	0,07
Calcium	24,4	28,8	20,7	0,003	0,09
Eisen	22,6	27,6	18,5	0,000	0,11
Vitamin B6	18,0	20,0	16,4	0,140	0,05
Omega-Fettsäuren (z. B. Omega‑3, Fischöl oder Algenöl)	17,6	23,3	12,9	0,000	0,13
Folsäure (Vitamin B9)	17,6	19,5	16,1	0,146	0,04
Vitamin B1	15,7	17,6	14,2	0,142	0,04
Vitamin B2	15,0	16,0	14,2	0,417	0,02
Biotin (Vitamin B7, Vitamin H)	14,5	17,6	11,8	0,008	0,08
Proteine	13,8	21,6	7,4	0,000	0,20
Vitamin A	12,8	13,4	12,3	0,599	0,02
Vitamin E	12,7	12,7	12,8	0,981	0,00
Kalium	12,2	14,5	10,2	0,038	0,06
Selen	12,1	13,5	11,0	0,218	0,04
Vitamin K	11,7	13,4	10,3	0,129	0,05
Jod	11,6	13,0	10,5	0,204	0,04
Melatonin	11,4	13,3	9,8	0,082	0,05

Gesamt: *n* = 1071, SoMe-Informierte: *n* = 486, Nicht-SoMe-Informierte: *n* = 585 (Fallzahlen ungewichtet)

*NEM* Nahrungsergänzungsmittel, *SoMe* soziale Medien

Die Einnahme eines Kombinationspräparats mit mehreren Inhaltsstoffen gaben 40,6 % der NEM-Nutzenden für die letzten 12 Monate an. Weitere 15,8 % waren sich diesbezüglich unsicher.

Nach der Häufigkeit der Einnahme gefragt, gaben 29,6 % der NEM-Nutzenden an, im letzten Jahr täglich NEM eingenommen zu haben. Weitere 33,4 % berichteten eine mindestens wöchentliche Einnahme.

Mit Blick auf die zukünftige Einnahme schätzte es etwa ein Viertel aller Befragten (24,6 %) als eher oder sehr wahrscheinlich ein, in den kommenden 12 Monaten ein NEM einzunehmen, das sie bisher noch nicht eingenommen hatten. Eine Mehrheit (58,1 %) hielt dies hingegen für eher oder sehr unwahrscheinlich.

### Wahrnehmung, Einnahmemotivation und Risiko-Nutzen-Einschätzung

Bei der Bewertung verschiedener Aussagen zu NEM stimmten 63,2 % der Befragten zu, dass NEM für gesunde Menschen mit ausgewogener Ernährung nicht für die Versorgung mit Nährstoffen notwendig sind. Gleichzeitig schrieben 36,5 % der Befragten NEM auch für gesunde Menschen mit ausgewogener Ernährung eine positive Wirkung zu. Mit Blick auf die Charakterisierung von NEM stimmten 57,7 % der Befragten der Aussage, dass NEM freiverkäufliche Arzneimittel seien, zu. Im Gegensatz dazu halten nur 14,3 % der Befragten NEM für Lebensmittel. Darüber hinaus gingen 46,6 % der Befragten davon aus, dass NEM auf ihre gesundheitliche Unbedenklichkeit geprüft werden, bevor sie in Deutschland verkauft werden.

**Abb. 1:**
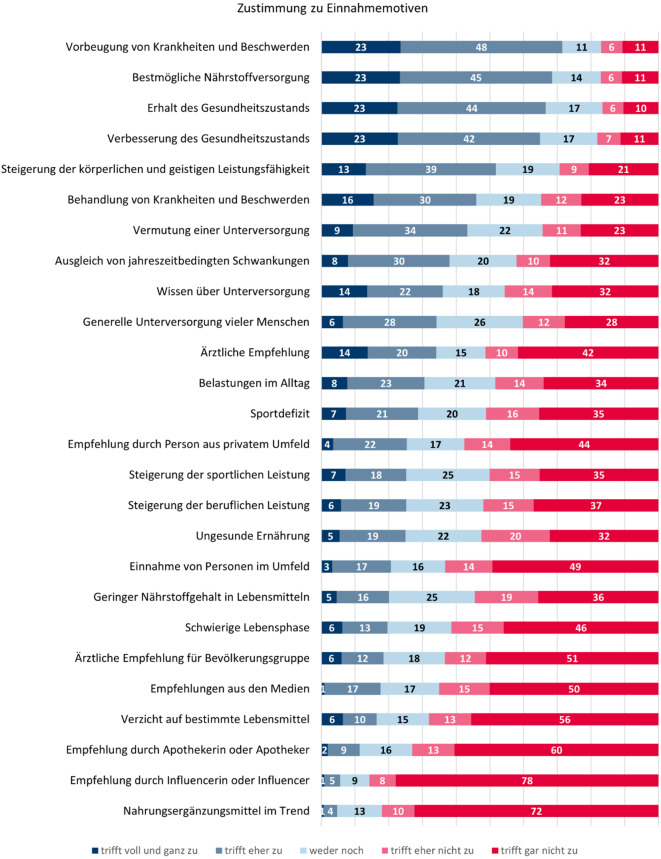
Zustimmung zu Motiven für die NEM-Einnahme unter NEM-Nutzenden; Angaben in Prozent; *n* = 824 (Fallzahl ungewichtet); Sortierung absteigend nach Zustimmung („trifft voll und ganz zu“ oder „trifft eher zu“); Rundungsdifferenzen möglich. NEM Nahrungsergänzungsmittel

Als Einnahmemotive wurden vorrangig solche genannt, die mit dem allgemeinen Gesundheitszustand assoziiert sind. Insbesondere der Vorbeugung von Krankheiten und der bestmöglichen Versorgung mit Nährstoffen stimmten die Befragten als Beweggründe für die Einnahme zu (Abb. 1). Weitere Verwendungsgründe waren der Erhalt oder die Verbesserung des eigenen Gesundheitszustands, aber auch die Behandlung von Krankheiten oder gesundheitlichen Problemen. Die Hälfte der NEM-Nutzenden gab die Steigerung der eigenen körperlichen oder geistigen Leistungsfähigkeit als Beweggrund für die Einnahme an. Weniger als die Hälfte der Befragten gab an, zu vermuten oder zu wissen, dass sie mit bestimmten Nährstoffen unterversorgt sind.

**Abb. 2:**
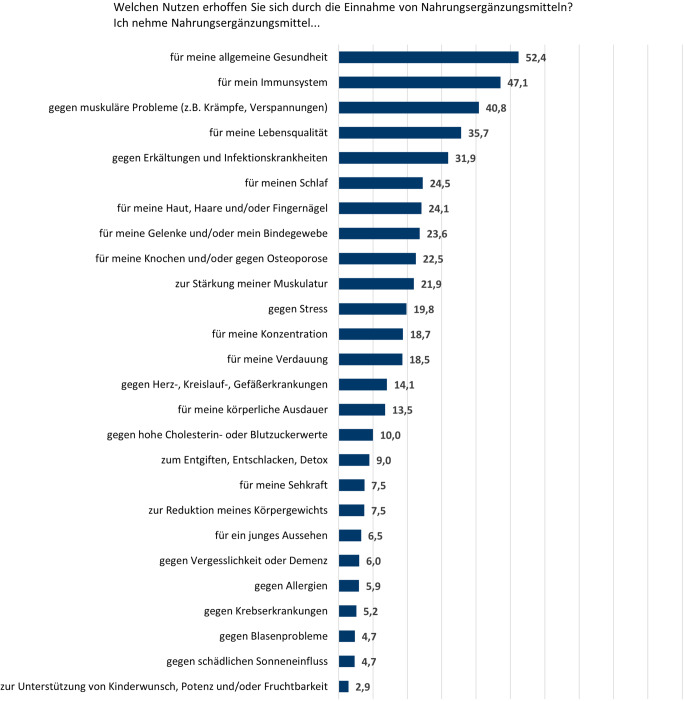
Anwendungsbereiche, in denen sich NEM-Nutzende durch die Einnahme von NEM einen Nutzen erhoffen; Angaben in Prozent; *n* = 824 (Fallzahl ungewichtet). *NEM* Nahrungsergänzungsmittel

Nach spezifischen Anwendungsbereichen gefragt, gab mehr als die Hälfte der NEM-Nutzenden an, sich von der Einnahme von NEM einen Vorteil für die allgemeine Gesundheit zu versprechen (Abb. 2). Des Weiteren erhofften sich viele Befragte einen Nutzen für ihr Immunsystem bzw. gegen Erkältungen und Infektionskrankheiten, gegen muskuläre Probleme und für ihre Lebensqualität.

**Abb. 3:**
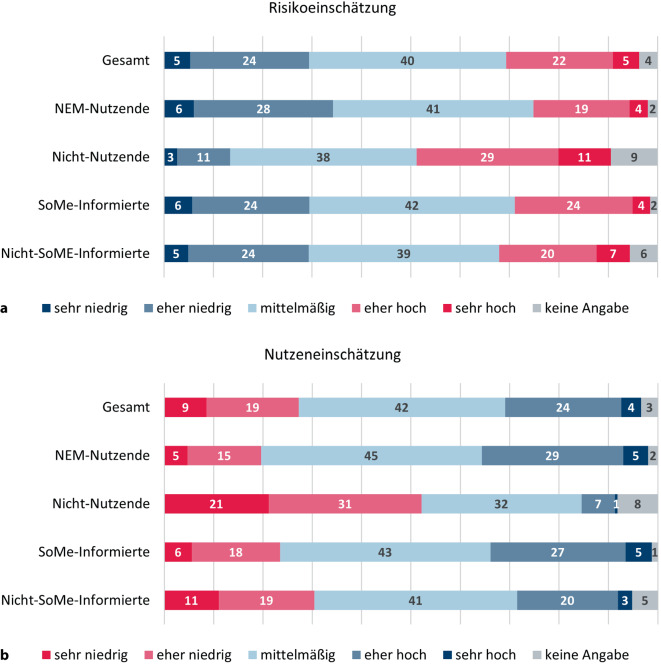
Risiko- (**a**) und Nutzeneinschätzung (**b**) nach NEM-Einnahme und SoMe-Informiertheit; Angaben in Prozent; gesamt: *n* = 1071, NEM-Nutzende: *n* = 824, Nichtnutzende: *n* = 247, SoMe-Informierte: *n* = 486, Nicht-SoMe-Informierte: *n* = 585 (Fallzahlen ungewichtet); Rundungsdifferenzen möglich. *NEM* Nahrungsergänzungsmittel, *SoMe* soziale Medien

In der allgemeinen Bevölkerung wurden das Risiko und der Nutzen bei der Einnahme von NEM ohne ärztliche Absprache im Durchschnitt gleichermaßen mittelmäßig eingeschätzt (Abb. 3). Nach Einnahme differenziert, schätzten NEM-Nutzende den Nutzen signifikant höher (M = 3,15, SD = 0,91 vs. M = 2,29, SD = 0,92; t(1033) = 12,14; *p* < 0,001; d = 0,94) und das Risiko signifikant niedriger (M = 2,86, SD = 0,93 vs. M = 3,37, SD = 0,94; t(1030) = 7,07; *p* < 0,001; d = 0,54) ein als Nichtnutzende.

### Subjektive Informiertheit

Über ein Drittel der Befragten fühlte sich gut oder sehr gut über Einnahmeempfehlungen (37,9 %) und empfohlene Höchstmengen (38,4 %) sowie den gesundheitlichen Nutzen (38,5 %) von NEM informiert. Im Vergleich sahen sich weniger Befragte hinsichtlich der gesundheitlichen Risiken (27,7 %) und der gesetzlichen Regelungen und Kontrollen (17,7 %) bei NEM gut oder sehr gut informiert.

### Rolle sozialer Medien

Insgesamt 45,4 % der Befragten zählten zu den SoMe-Informierten. Diese gaben an, insbesondere über die Plattformen Instagram (55,9 %), YouTube (42,7 %) und Facebook (39,2 %), aber auch TikTok (14,8 %) Informationen zu NEM wahrgenommen zu haben.

75,6 % der SoMe-Informierten nehmen nach eigener Angabe mindestens gelegentlich Informationen oder Werbung zu NEM von Influencerinnen und Influencern wahr. Deren fachlicher Hintergrund wurde dabei zumeist in den Bereichen Sport (34,1 %), Ernährungswissenschaft oder -beratung (23,1 %), Lifestyle-Coaching (20,1 %) und Medizin (13,0 %) verortet. Zudem gaben 30,9 % an, Informationen zu NEM von Influencerinnen und Influencern erhalten zu haben, denen sie keinen fachlichen Hintergrund zuschreiben. Bei Informationen von Influencerinnen und Influencern überwog zudem der Eindruck, dass insgesamt eher über Vorteile (59,1 %) als über Nachteile (2,3 %) oder neutrale Informationen (25,3 %) von NEM berichtet wird.

#### Nutzungsverhalten.

Im Vergleich zu den Nicht-SoMe-Informierten war der Anteil an NEM-Nutzenden bei SoMe-Informierten signifikant höher (80,0 % vs. 73,8 %; Χ^2^(1, 1070) = 5,40; *p* = 0,021), der Gruppenunterschied ist jedoch als sehr klein zu interpretieren (V = 0,07). Unter NEM-Nutzenden war die Anzahl eingenommener Stoffe bei den SoMe-Informierten (M = 8,77, SD = 8,27) signifikant höher als bei den Nicht-SoMe-Informierten (M = 6,88, SD = 7,30; t(822) = 3,39; *p* < 0,001; d = 0,24). Mit Blick auf einzelne Inhaltsstoffe zeigten sich für Proteine, Omega-Fettsäuren (z. B. Omega‑3, Fischöl oder Algenöl) und Eisen Unterschiede mit signifikant höherer Einnahme unter den SoMe-Informierten im Ausmaß eines kleinen Effekts (Tab. 2).

Verglichen mit Nicht-SoMe-Informierten war der Anteil der SoMe-Informierten, der mindestens einmal in der Woche NEM einnimmt, signifikant höher (55,2 % vs. 42,4 %; Χ^2^(1, 1070) = 16,60, *p* < 0,001, V = 0,12). Zudem hielten es SoMe-Informierte für wahrscheinlicher, in den kommenden 12 Monaten ein NEM einzunehmen, das sie bisher noch nicht eingenommen hatten (M = 2,69, SD = 1,26; t(1053) = 6,18, *p* < 0,001, d = 0,39), als Nicht-SoMe-Informierte (M = 2,20, SD = 1,26).

#### Wahrnehmung.

Bei der Wahrnehmung von NEM zeigte sich insbesondere bei einer Aussage ein Gruppenunterschied: SoMe-Informierte stimmten signifikant stärker zu (M = 3,23, SD = 1,12) als Nicht-SoMe-Informierte (M = 2,91, SD = 1,09), dass NEM für gesunde Menschen mit ausgewogener Ernährung eine positive Wirkung haben (t(1049) = 4,54, *p* < 0,001, d = 0,29).

#### Einnahmemotivation.

**Abb. 4:**
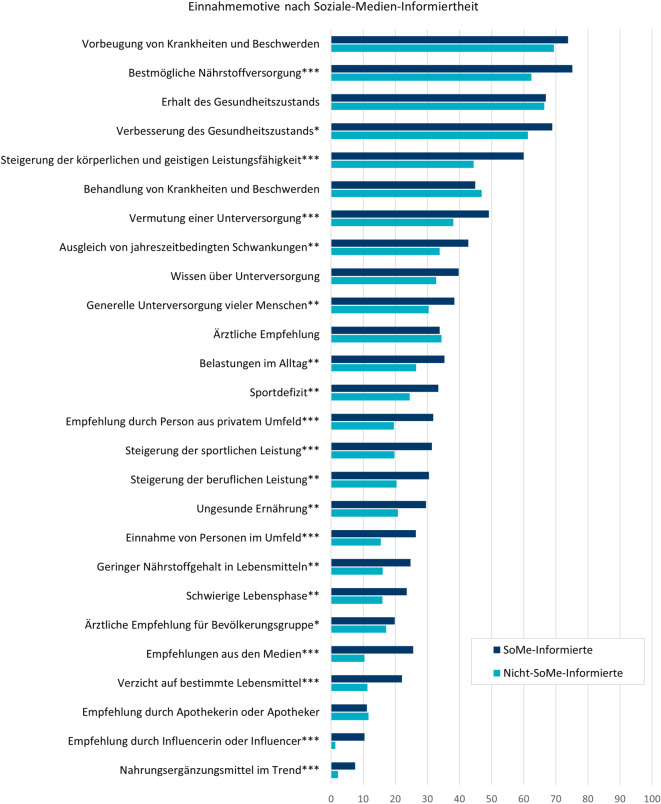
Zustimmung zu Motiven für die NEM-Einnahme („trifft voll und ganz zu“ oder „trifft eher zu“) unter NEM-Nutzenden nach SoMe-Informiertheit; signifikante Mittelwertunterschiede: *p < 0,05, **p < 0,01, ***p < 0,001; Angaben in Prozent; SoMe-Informierte: *n* = 391, Nicht-SoMe-Informierte: *n* = 433 (Fallzahlen ungewichtet). *NEM* Nahrungsergänzungsmittel, *SoMe* soziale Medien

Alle Einnahmemotive gemittelt, stimmten SoMe-Informierte diesen signifikant stärker zu (M = 2,70, SD = 0,63; t(822) = 5,93, *p* < 0,001, d = 0,42) als Nicht-SoMe-Informierte (M = 2,43, SD = 0,66). Auch für einzelne Motive zeigten sich Unterschiede: Beispielsweise stimmten SoMe-Informierte im Vergleich zu Nicht-SoMe-Informierten eher zu, NEM aufgrund des Wunsches einer körperlichen oder geistigen Leistungssteigerung einzunehmen (t(822) = 4,92, *p* < 0,001, d = 0,35; Abb. 4).

#### Anwendungsbereiche.

Bei den Anwendungsbereichen zeigten sich für die Bereiche Lebensqualität, Haut, Haare und Nägel und Stress Unterschiede zwischen SoMe-Informierten und Nicht-SoMe-Informierten im Ausmaß eines kleinen Effekts. SoMe-Informierte erhofften sich in diesen Bereichen signifikant häufiger einen Nutzen (Χ^2^s ≥ 14,79, *p*s < 0,001, Vs ≥ 0,12).

#### Risiko-Nutzen-Einschätzung.

Differenziert nach SoMe-Informiertheit zeigte sich auch in der Nutzeneinschätzung ein signifikanter Unterschied (t(1033) = 4,07, *p* < 0,001, d = 0,26; Abb. 3): SoMe-Informierte schätzten den Nutzen höher ein (M = 3,09, SD = 0,89) als Nicht-SoMe-Informierte (M = 2,83, SD = 0,98). Bei der Risikoeinschätzung zeigte sich kein Unterschied (t(1030) = 0,52, *p* = 0,601, d = 0,03).

#### Subjektive Informiertheit.

Insgesamt wurde die subjektive Informiertheit über NEM von SoMe-Informierten (M = 3,10, SD = 0,90) signifikant höher eingeschätzt als von Nicht-SoMe-Informierten (M = 2,89, SD = 0,93; t(971) = 3,62, *p* < 0,001, d = 0,24).

## Diskussion

Da NEM bei Verbraucherinnen und Verbrauchern sowie in sozialen Medien sehr beliebt sind, liefert die beschriebene Befragung wichtige Erkenntnisse darüber, wie Menschen in Deutschland diese Produkte nutzen, welche Gründe sie dafür haben und wie sie diese wahrnehmen. Zudem beleuchtet die Befragung die Bedeutung sozialer Medien im Zusammenhang mit NEM.

Mit über 3 Viertel der Befragten, die angaben, in den vergangenen 12 Monaten mindestens einen Nähr- oder Inhaltstoff über NEM eingenommen zu haben, entsprechen die Ergebnisse etwa denen einer aktuellen Studie [2] und weisen auf eine hohe Popularität der NEM-Einnahme hin. Während weitere Studien die Nutzungsprävalenz über das Einkaufsverhalten bestimmt haben [1, 27], liefert die vorliegende Studie Erkenntnisse zum Einnahmeverhalten. Verglichen mit der 2008 veröffentlichten Nationalen Verzehrsstudie II, in der eine Einnahmeprävalenz von etwa 28 % berichtet wird [28], bestätigt sich ein Anstieg der Nutzung.

Die Abfrage der NEM-Nutzung auf Stoffebene in der vorliegenden Studie bietet zudem Vorteile gegenüber einer globalen Einschätzung der NEM-Nutzung. Zum einen deuten Diskrepanzen zwischen selbstberichtetem und beobachtetem Einkaufsverhalten hinsichtlich NEM darauf hin, dass Befragte Schwierigkeiten haben, ihre allgemeine NEM-Nutzung korrekt einzuschätzen [27]. Die beschriebene detaillierte Abfrage ermöglicht dabei eine präzisere Schätzung der Prävalenz. Zum anderen kann die Einnahme einzelner Nähr- und Inhaltsstoffe differenziert betrachtet werden.

So zeigt sich, dass insbesondere einzelne Mikronährstoffe wie Magnesium oder Vitamin D über NEM eingenommen werden. Durchschnittlich gaben die Befragten an, fast 8 verschiedene Nähr- und Inhaltstoffe über NEM einzunehmen. Dabei ist die Einnahme von Kombinationspräparaten mit mehreren Nähr- und Inhaltsstoffen zu berücksichtigen, die über 40 % der Befragten bestätigten.

Bei der Wahrnehmung von NEM zeigte sich, dass sich die rechtliche Definition von NEM nicht mit dem Verständnis der Verbraucherinnen und Verbraucher deckt. So werden NEM eher als freiverkäufliche Arzneimittel und nicht entsprechend der Nahrungsergänzungsmittelverordnung als Lebensmittel angesehen. Auch teilen trotz fehlender Zulassungspflicht für NEM viele die Annahme, dass NEM auf gesundheitliche Unbedenklichkeit geprüft werden. Obwohl die Popularität von NEM stark gestiegen ist, scheinen Kenntnisse zur Regulierung weiterhin kaum verbreitet, wie es bereits in früheren Studien gezeigt wurde [27, 29]. Gleichzeitig scheint man sich des fehlenden Wissens bewusst zu sein, denn der Großteil der Befragten in der aktuellen Studie fühlt sich nicht gut über gesetzliche Regelungen und Kontrollen bei NEM informiert.

Übereinstimmend mit Ergebnissen aus den Niederlanden [30] zeigen die aktuellen Ergebnisse auch für Deutschland, dass Verbraucherinnen und Verbraucher, die NEM einnehmen, den Nutzen davon für größer und das Risiko für geringer halten als diejenigen, die keine NEM einnehmen. Andere Studien weisen darauf hin, dass insbesondere der wahrgenommene Nutzen für die Einnahmeentscheidung von Bedeutung ist [31]. Dass der gezeigte Gruppenunterschied zwischen NEM-Nutzenden und Nichtnutzenden für die Nutzeneinschätzung höher ausfällt, deutet ebenfalls darauf hin. Die ausgeprägte Nutzenwahrnehmung zeigt sich auch in der Annahme, dass NEM auch für gesunde Menschen mit ausgewogener Ernährung eine positive Wirkung haben, die mehr als ein Drittel der Befragten in der vorliegenden Studie teilt.

Das Nutzenverständnis spiegelt sich zudem in den Einnahmemotiven wider, die vor allem von präventiven gesundheitsbezogenen Motiven geprägt sind. Die Verbesserung und der Erhalt der Gesundheit wurden bereits zuvor als zentrale Motive bei der Einnahme von NEM berichtet [32]. Darüber hinaus deuten Forschungsergebnisse darauf hin, dass gesundheitsbewusste Menschen eine positivere Einstellung zu NEM haben [33]. Die vorliegende Studie verdeutlicht zusätzlich auch die Relevanz von sozialen oder leistungsbezogenen Motiven.

Die Studienergebnisse zeigen Unterschiede zwischen SoMe-Informierten und Nicht-SoMe-Informierten im Nutzungsverhalten, in der Einnahmemotivation und in der Wahrnehmung von NEM. Im Vergleich mit Nicht-SoMe-Informierten geben SoMe-Informierte an, eine höhere Anzahl verschiedener Stoffe über NEM einzunehmen und NEM eher mindestens wöchentlich zu verwenden. Sie halten es darüber hinaus für wahrscheinlicher, zukünftig NEM mit weiteren Stoffen auszuprobieren. Zudem schreiben SoMe-Informierte NEM einen höheren Nutzen zu. Dies schlägt sich nicht nur in der globalen Nutzeneinschätzung, sondern auch in der Bewertung spezifischer Motive zur Einnahme von NEM nieder.

Beispielsweise verwenden SoMe-Informierte NEM eher, um die sportliche Leistung zu steigern. Zudem erhoffen sie sich häufiger einen Nutzen für ihre Haut, Haare und Nägel. Diese Aspekte, die mit einem sportlichen Lebensstil oder einem idealen Körperbild verknüpft sind, werden auch in (Werbe‑)Inhalten von Influencerinnen und Influencern aufgegriffen [15, 34].

Die Unterschiede zwischen den Gruppen lassen vermuten, dass die Versprechen, mit denen NEM in sozialen Medien beworben werden, die Ansichten und das Verhalten von SoMe-Nutzenden beeinflussen könnten. Dies wird dadurch gestützt, dass Werbung für NEM in sozialen Medien häufig die Vorteile dieser Produkte hervorhebt und Informationen über Risiken unerwähnt bleiben [35]. Die vorliegenden Ergebnisse zeigen zudem, dass sich dies auch in der Wahrnehmung der SoMe-Nutzenden widerspiegelt.

Trotz der festgestellten Gruppenunterschiede begründen NEM-Nutzende ihre Einnahme nicht mit der Empfehlung durch Influencerinnen und Influencer. Denkbar ist, dass sich die im Rahmen einer Empfehlung genannten (Werbe‑)Aussagen in nutzenspezifischen Einnahmemotiven niederschlagen, die Empfehlung selbst als Informationsquelle, nicht aber als Grund für die Einnahme gesehen wird. Darüber hinaus ist es möglich, dass ein potenzieller Einfluss von Informationen in sozialen Medien auf Wahrnehmung und Verhalten von Betroffenen selbst unterschätzt wird. So zeigen Studien einen Third-Person-Effekt, bei dem Menschen den Einfluss von Werbung auf sich selbst geringer einschätzen als auf andere Personen [36–38]. Die gezeigten Unterschiede werfen folglich die Frage auf, inwiefern diese in der Exposition mit Informationen in sozialen Medien oder in potenziellen Drittvariablen begründet sind.

Dass fast die Hälfte der Befragten mindestens gelegentlich über soziale Medien Informationen zu NEM wahrnimmt, zeigt die Bedeutung des Mediums für die Gesundheitskommunikation zu NEM. Ein Einfluss von NEM-Werbung in sozialen Medien auf die Wahrnehmung und Nutzung von NEM würde zusätzliche Implikationen für die Risikokommunikation bedeuten. So besteht die Gefahr, dass bei der Bewerbung von NEM durch Influencerinnen und Influencer ein verzerrtes Bild gezeichnet wird. Neben der Verwendung von Gesundheitsversprechen, die in Teilen als unzulässig einzustufen sind [17], werden beispielsweise auch Höchstmengenempfehlungen für die Einnahme und Angaben über mögliche Risiken kaum kommuniziert [35]. Verbraucherinnen und Verbraucher sind allerdings vom Erhalt solcher Informationen abhängig, um informierte Konsumentscheidungen zu treffen. Dafür braucht es wissenschaftlich fundierte und unabhängige Informationsangebote, auch in sozialen Medien.

### Limitationen und zukünftige Forschung

Bei der Interpretation der vorliegenden Daten müssen limitierende Faktoren berücksichtigt werden. Zum einen basieren die vorliegenden Daten auf Selbstberichten. Diese sind bei retrospektiven Angaben (z. B. zur NEM-Einnahme in den vergangenen 12 Monaten) von der Erinnerung der Befragten abhängig und damit anfällig für Verzerrungen. Ein valider Selbstbericht über die NEM-Einnahme setzt zudem voraus, dass die Befragten über das notwendige Wissen verfügen, um Produkte (nicht) als NEM zu kategorisieren. In Anbetracht der verbreiteten Wahrnehmung von NEM als Arzneimittel und der vermarktungsbedingten Ähnlichkeiten beider Produktgruppen kann trotz der in der Frage enthaltenen Definition nicht ausgeschlossen werden, dass einzelne Befragte fälschlicherweise auch die Einnahme von Inhaltsstoffen über freiverkäufliche Arzneimittel angegeben haben.

Zum anderen lässt das Querschnittsdesign der Studie keine Schlussfolgerungen über Wirkrichtung oder Kausalität zu. Es bleibt zu klären, inwieweit die gezeigten Unterschiede zwischen SoMe-Informierten und Nicht-SoMe-Informierten auf die Wirkung von Inhalten und Werbung zu NEM in sozialen Medien zurückzuführen sind oder von konfundierenden Variablen abhängen. Auch ist möglich, dass NEM-Nutzende häufiger Informationen zu NEM in sozialen Medien begegnen, weil sie aktiv danach suchen. Dementsprechend ist weitere Forschung notwendig, um den Verarbeitungsprozess von Informationen zu NEM in sozialen Medien tiefer zu untersuchen.

Dabei bilden junge Menschen eine wichtige Zielgruppe, die aufgrund der kleinen Fallzahl in dieser Studie nicht detailliert beleuchtet werden kann. Influencerinnen und Influencer in sozialen Medien spielen für die Meinungsbildung Jugendlicher eine wichtige Rolle [39, 40]. Darüber hinaus nehmen soziale Medien auch auf die Konsumentscheidungen junger Menschen Einfluss. So geben 35 % der Jugendlichen an, bereits ein Produkt nach der Empfehlung einer Influencerin oder eines Influencers gekauft zu haben [40]. Gleichzeitig werden bereits im jugendlichen Alter NEM konsumiert [41]. Daher braucht es Forschung, die spezifisch das Informationsverhalten und die Motive für die NEM-Einnahme von jungen Menschen untersucht, um eine zielgruppengerechte Risikokommunikation zu ermöglichen.

## Fazit

Die Einnahme von Nahrungsergänzungsmitteln (NEM) ist weitverbreitet und insbesondere mit gesundheitsbezogenen Motiven verbunden. Gleichzeitig schlagen sich regulatorische Aspekte nur bedingt in der Wahrnehmung der NEM-Nutzenden nieder. Die Tatsache, dass sich die Nutzung und die Gründe für die Einnahme von NEM in Abhängigkeit davon unterscheiden, ob Menschen Informationen darüber über soziale Medien erhalten, deutet darauf hin, dass die Art und Weise, wie diese Produkte in sozialen Medien beworben werden, einen Einfluss auf Verbraucherinnen und Verbraucher haben könnte. Die aktuelle Studie zeigt dabei die Relevanz von sozialen Medien für die Gesundheits- und Risikokommunikation sowie den Bedarf für weitere Forschung auf.

## Data Availability

Die während der vorliegenden Studie erzeugten und/oder analysierten Datensätze sind auf begründete Anfrage bei der Korrespondenzperson erhältlich.

## Footnotes

1 Kriterien für den Ausschluss waren Befragungszeiten < 40 % des Medians, fehlende Varianz (SD = 0) bei der Abfrage der Einnahmemotive sowie implausible Antworten in offenen Angaben.
